# Perspectives of Caring for Older Persons: A Scoping Review

**DOI:** 10.1177/21501319241296618

**Published:** 2024-11-14

**Authors:** Ignatius Chida, Manohar Pawar, Ndungi Mungai

**Affiliations:** 1 Charles Sturt University, Dubbo, NSW, Australia; 2 Charles Sturt University, Wagga Wagga, NSW, Australia

**Keywords:** aging, caring for the elderly population, formal and informal care, home-based care, multiple care perspectives

## Abstract

In this article we explore caring practices for older persons from multiple care perspectives related to organizing home-based care. We employed a scoping review method and searched 5 electronic database using relevant key words and identified 62 articles for codebook thematic analysis. Our analysis identified 6 key themes in older people’s care: gender issues; socio-economic status; psychological; cultural issues; elder abuse; and legal, ethical, and human rights concerns. Findings show that despite notable research progress made in recent years on home-based care for older people, research gaps still exist. Researchers and practitioners are encouraged to consider viewing home-based care for older people from multiple perspectives to ensure a holistic understanding of an individual’s needs and circumstances and organize care accordingly. Future research and practice should seek to understand the lived experiences of care givers and receivers from multiple care perspectives, to help inform responsive and effective home-based care policies and programs.

## Introduction

In the context of home-based care, in this article we discuss caring practices for older persons from multiple care perspectives and complexities in and around the issues relating to organizing care. From the current scoping review, “perspectives” refers to 6 main themes confirmed in the literature and our codebook thematic data analysis, including gender, socio-economic status, psychological, cultural issues, elder abuse, and legal, ethical and human rights perspectives. It may be clarified at the outset that the terms, “care dimension” and “care perspective” are used interchangeably in this article. In the current evidence, care perspectives such as gender, social, economic, and cultural factors influence care provision for older people living at home.^1^ In a 2020 report, the World Health Organization^1^ further offers a targeted global health policy directive under “Areas for action 3.4” calling on member-states to ensure high quality long-term care is provided to older people to maintain their functional ability, thus aging with dignity and respect of human rights. There is no universally agreed-upon definition of older persons. In this scoping review, older persons can be defined as anyone aged 60 years or over.^2^ Informed policy making and planning require an understanding of these caring perspectives and their influence on care situations for older persons living at home.

As approaches to caring for older people living at home continues to evolve, understanding, and adapting to the associated changing care dynamics is imperative. In this article, we therefore seek to examine how the aforementioned care perspectives contribute individually and collectively to our holistic understanding of home-based care for older people. This scoping review follows this logic and sequencing. That is, the 6 care perspectives are examined individually prior to analyzing them thematically to gain a holistic understanding of the research to date. These 6 caring perspectives are delineated in terms of features and nature, research gaps, and issues. In previous caregiving literature, the focus was largely on the influence of atomistic care dimensions in understanding home-based care situations for older people. The term atomistic care in prior research refers to a consistent, individualistic approach to understanding older persons’ life circumstances and preferences that potentially influence their home-based care situations, rather than a holistic approach that considers both the individual and societal influences.^3^ This scoping review draws on this research while also using an holistic and integrated approach that combines the aforementioned 6 older people’s care perspectives that we suggest is needed to ensure high quality of care for older persons. In the context of this scoping review, atomistic care refers to a fragmented or piecemeal approach to providing care, where individual tasks or aspects of care are addressed in isolation rather than as part of a comprehensive, coordinated strategy. It should be noted that the influences of care perspectives vary among older persons based on their unique homecare circumstances.

### Atomistic Care

The global population is aging rapidly because people are now living longer than a century ago and there is a decline in fertility.^4^ Further projections of this trend suggest that globally, by 2050, the world population of older adults aged 65 years and over will rise from 761 million in 2021 to approximately 1.6 billion by 2050 when the majority of older people will live in their own homes and communities, rather than in residential care facilities.^5^ As a result, home-based care issues are becoming topical with this changing demographic profile, especially regarding the best care practices to meet home-based needs of this group. Additionally, research^6-8^ has indicated that despite significant funding investments made particularly in many developed countries, complex home-based care issues, and unmet care needs remain major concerns.

A limitation of previous research in this area is that it followed an atomistic approach to understanding caring needs of older persons living in their own homes. To date, there has been no scoping review conducted examining the complexities of these issues. In addition, little attention has been paid to understanding home-based care for older people from multiple care perspectives. For example, in previous studies, various authors have reported the varying ways that gender issues might influence the care situations of older persons living in the home.^9-12^ Additionally, other authors also found in a cluster of studies that socio-economic status^13-15^ and psychological issues^16-18^ may influence the older people’s homecare care experiences. Furthermore, researchers have demonstrated that cultural issues play a significant role in the homecare support circumstances for older persons receiving care.^19-21^ Findings from a recent Australian study^22^ demonstrated that issues of elder abuse may become prevalent during the older person’s care at home, especially in situations where issues related to wills and inheritance are involved. Moreover, in countries such as Australia, home-based older persons’ rights to personal property and finances may be legally protected through an enduring power of attorney (POA).^23^

In another cluster of studies, it has been demonstrated that the home-based care landscape for older people is fraught with a combination of challenges, including rising high health care costs,^24^ shortage of care staff,^25^ high burden of diseases,^26^ and insufficient aged care funding.^7^ Caregiving literature to-date include disparate care perspectives for older people living in the home. To improve home-based care, we recommend a more holistic approach, where care providers consider the entire context of a patient’s needs and work collaboratively to provide integrated, patient-centered care. We suggest that analyzing and addressing the 6 perspectives in an integrated manner has the potential to promote quality, holistic care for older persons.

Thus, the objectives of this scoping review are:

(1) to synthesize evidence from the literature about a range of care perspectives for older persons; and
(2) to describe strengths, gaps, and issues in each of the identified care perspective and discuss inherent implications for policy and practice.

## Methods

### Rationale

This article followed^27^ scoping review methodological framework as discussed below.

### Stage 1: Identifying the Research Question

The following key research questions guided our scoping review:

(1) What is known from the literature about the range of care perspectives related to organizing home-based care for older persons?
(2) What are the strengths, research gaps and issues in each of the identified care perspectives and the implications for policy and practice?

### Stage 2: Identifying Relevant Studies

As presented in Table 1, in consultation with a senior research librarian, 5 databases and 13 relevant keywords/search strings were used to identify relevant articles in this scoping review. The scoping review used the inclusion and exclusion criteria to identify relevant studies (see Table 2). Relevant gray literature was additionally included in this study but comprised only a very minimal proportion of the total number of manuscripts included. Secondly, the relevant identified manuscripts were hand-searched to identify any missing studies from the traditional search as recommended in the literature.^27^ The process of identifying suitable articles was underpinned by initial title and abstract screening. Then, if the abstract appeared to meet the criteria, it was reviewed. Subsequently, if an abstract that was deemed suitable, then full text reading of the article was done to further scrutinize it for inclusion or exclusion.

**Table 1.** Databases and Database Keywords.

Database	Database keywords
EbscoHost databases—Academic Search Complete, CINAHL Plus with Full Text, Humanities International Complete, Psychology and Behavioral Sciences Collection, SocINDEX with Full Text	Caregiv* AND “OPPORTUNITY costs” AND “Informal Care”
Proquest	noft(“socioeconomic status”) AND noft(“informal care”) AND noft(“older people”)
Taylor and Francis	Family AND “Socioeconomic Inequalities” AND “Informal Caregiving”
Proquest	noft(“socioeconomic status”) AND noft(“loneliness”) AND noft(“older people”)
EbscoHost databases—Academic Search Complete, CINAHL Plus with Full Text, SocINDEX with Full Text.	“caregiver burden” AND “psychological distress” AND centenarians OR “oldest old people” NOT “nursing home or long term care facility”
Scopus	Depression AND Caregivers AND “Frail Older adults”
Proquest	noft("psychological well-being") AND noft(caregivers) AND noft("older people")
EbscoHost databases—CINAHL Plus with Full Text, Humanities International Complete, Psychology and Behavioral Sciences Collection, SocINDEX with Full Text	“Elder abuse” AND Caregivers AND “Risk factors”
Taylor & Francis	“Older people” AND “financial Abuse” AND “Family members”
Proquest	noft(caregiver) AND noft(“gender inequalit*”) NOT noft(Children) AND noft(parent)
Oxford Academic	“Gender differences” AND “Spousal caregiving” AND “Older people”
EbscoHost databases—Academic Search Complete, CINAHL Plus with Full Text, Humanities International Complete, Psychology and Behavioral Sciences Collection, SocINDEX with Full Text	Gender AND Caregiver AND “Older people” AND “Adult children”
Oxford Academic	“Filial responsibility” AND “family members” AND “older people” AND Roles AND culture

**Table 2.** Exclusion and Inclusion Criteria.

Criteria	Inclusion	Exclusion
Language	English language	Published in a language other than English
Timeframe	2013-2023	Before 2013; after 2023
Article type	Peer-reviewed; non-peer-reviewed report	Non-peer-reviewed articles
Article focus	Articles concerned with home-based care for older people	Articles not examining aging and home-based care
Types of studies	Research studies using either qualitative or quantitative methods; Mixed-methods studies	Non-empirical literature
Settings	Home-based care support for older people	Formal care support for older people, for example, nursing homes, hospitals

### Stage 3: Selecting Studies

To ensure consistency, the initial retrieval process of manuscripts for inclusion and exclusion based on title and abstract was performed by the first author. The first author read fully all the selected eligible articles, closely checking the articles’ contexts and eligibility in terms of inclusion and exclusion criteria. To minimize chances of errors and biases, the second author independently verified and evaluated selected articles by reading entire texts for eligibility. Any disagreements between the 2 authors were discussed and resolved until consensus was reached in consultation with the third author. Consistent with scoping reviews, critical appraisal of included sources of evidence was not conducted.^28^

### Stage 4: Charting the Data and Coding

A codebook thematic analysis approach^29^ utilizing Nvivo software for data extraction and management was used. The codebook thematic analysis was chosen because of its key functionalities including allocation of data to predetermined themes.^29,30^ Research evidence^29,30^ argued that the codebook thematic analysis is a pragmatic approach that combines the development of codes and themes/categories. It involves having to read and re-read each of the publication included to familiarize with the evidence emerging. We followed this by drawing codes from the data which we then further deductively used to code any remaining data, while constantly being open to new emerging codes and themes.

The first author completed the initial data charting and coding. The 3 authors then discussed the outcomes of the final data coding and reached consensus on the themes/categories that emerged from the analysis of the data. Nvivo software was used to sort and manage the data into codes. We followed an iterative process to refine themes and clustering them into categories.^29^ To achieve transparency, in this review, we applied key specific terms and a comprehensive search of the topic.^29^ The 6 themes in the literature were then confirmed by the 3 authors.

### Stage 5: Collating, Summarizing and Reporting Research Findings

Following meticulous review and extraction process of the required information, we then analyzed and analyzed the results from the extraction. To synthesize all relevant data, in this study, we utilized the following criteria: author, year of publication, method/instrument, place of origin and the sample, to develop a data-extracting chart (see Appendix 1). Due to the large, complex and heterogenous nature of the manuscripts included in this scoping review, we used a codebook thematic analysis approach. The codebook thematic analysis synthesized the data for each of 6 themes confirmed in the literature. We discuss the results below.

## Findings

### Manuscripts Included in This Review

**Figure 1.**
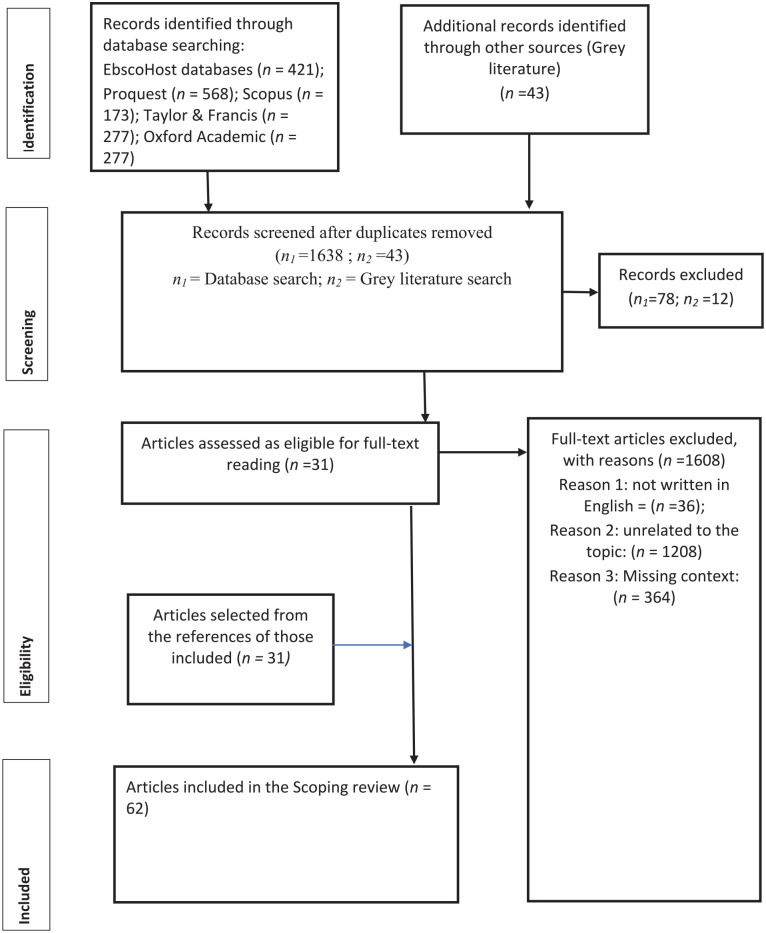
PRISMA flowchart.

Figure 1 below lists the manuscripts selected for inclusion in this review. The flowchart shows that 1716 articles were identified for screening, and potential selection for analysis. After the inclusion and exclusion criteria were applied, 31 manuscripts were identified for retrieval. Coincidentally, a further 31 manuscripts were identified for retrieval after hand searching the reference lists in the first 31 manuscripts included. The resulting 62 articles, as can be seen from Appendix 1, represented a diverse range of approaches, including quantitative, qualitative and mixed methods empirical research as well as literature reviews and document analyses.

#### Theme 1: Gender skewed care

The findings of caregiving research of the reviewed studies have consistently shown that traditionally women have been the main formal and informal caregivers. Some studies found that women, particularly partners or adult daughters, disproportionately take on a heavier care work burden than men.^11,12,31-34^ For example, older parents in need of care are most likely to be looked after by their daughters and not their sons. Evidence suggested that traditionally women are socialized into the care role and internalize the gender norms, which view care roles as women’s work.^10^ Moreover, researchers from Netherlands found that when considering the social role theory, women are viewed by several societies as the providers of much of the care.^12^

An emergent body of literature in caregiving notes a shift in care dynamics toward men contributing to care and this is beginning to gain scholarly attention. For example, an Australian-based systematic literature review^35^ acknowledges that, owing to feminist movements, more women have been able to join the work force, thereby contributing to household incomes. In addition, a report on migration^36^ shows that because of migration, for example in Latin America because of harsh economic conditions, women have been forced to search for economic opportunities overseas, leaving behind their husbands with children, and their older parents. Furthermore, a study on the difference between men and women in caregiving^34^ found that unlike women who traditionally provide personal care, men tend to offer non-personal and physical support, such as doing the gardens, cleaning the house, or helping with shopping. The study goes further to conclude that unlike men, women are more likely to be expected to provide personal care and spend more time caring.

Finally, in spousal relationships, women are more likely to provide informal care to their male partners. A quantitative study utilizing a survey method that involved 1611 participants (informal spousal caregivers), observed that men usually marry younger wives, and with age being a predictor of aging issues, women are more likely to be able to provide care for their husbands.^12^ Similarly, a European bibliographical review from Spain points out that older persons’ care responsibilities mainly fall on women.^31^ Research on spousal caregivers found that they rarely receive any support from members of their family, friends, or even formal health care professionals.^37^ While the gender skewed caring is a critical issue, it is useful to explore the complexity of the caring phenomena beyond this binary of men versus women.

#### Theme 2: Socio-economic status

Caregiving differs in many ways for both the caregiver and the care recipient, and more specifically based on their socio-economic status (SES). In relation to care needs, previous studies have shown that people from lower SES have a higher propensity for receiving and giving informal care,^38,39^ thus the likelihood of household members from low SES to provide care for their older loved ones is also higher. Older people from a higher SES are less likely to rely on or use informal care provided by family, opting to use their own resources to purchase formal care.^39,40^ As a result, their families may provide only minimal, if any, informal care support for them. In addition, unlike higher SES background, older people and their families who are reluctant to accept the concept of informal care, low SES families tend to hold strong caregiving cultural norms that are open to receiving or providing informal care.^38,41^ Thus, low SES status may be associated with informal care provision and uptake. Being unable to engage in paid employment owing to caring roles may push them into even lower SES.

Informal care provision is closely correlated with implications on the employment obligations of the caregiver. Some studies found that caregivers with more employment obligations find it hard to find the time to fulfill their care obligations.^14,42^ As a result, many caregivers, in particular, women end up giving up fulltime employment, opt for casual employment or reduce their work hours, thus negatively affecting their income and social status.^42,43^ Furthermore, research findings suggest that caregivers from low SES background, in comparison with those from higher SES, experience serious financial challenges and lower life satisfaction owing to the caring roles.^14,42,44,45^

Overall, research shows that informal care is greatly undervalued both politically and in economic terms,^46^ although its true economic value and contribution has been shown to be enormous to the Australian economy, for example. A report on the value of informal care in Australia estimated the total replacement cost for informal care in 2020 was $77.9 billion.^47^

#### Theme 3: Psychological issues

Recent research has shown that while caregiving may bring positive rewards and satisfying experiences for the caregiver^48^ it can also be a source of stress and raises some psychological issues.^16,17,49,50^ This is exemplified in a qualitative study, involving 112 participants (68 women and 44 men), which examined depression levels among primary caregivers of Ultra-Orthodox Jewish background.^16^ The research demonstrated that being both a carer and a spouse can present as high-risk factors for psychological problems among caregivers. Furthermore, being a caregiver for someone with special home-based care needs often places a high level of care burden on the caregiver. Evidence further discussed that caregivers of older people with complex care needs not only find balancing care responsibilities with work-life-family and other obligations extremely difficult to juggle,^51^ but more importantly, they experience considerable psychological stress and anxiety.^18^

Even though home-based care may have satisfying outcomes for both the caregiver and the care recipient, it has been noted that caregiving often presents double challenges particularly in cases where an older family member may be providing the care.^48^ In such an instance, the older family caregiver mostly a spouse, could be faced with their own personal aging care issues, but then also be expected to provide care to loved ones.^37^ In addition, research shows that lack of opportunity to participate in employment and the resultant loss of income because of caring can be a source of psychological distress for some informal caregivers.^17^ Adding on to these findings, it has been observed that older spousal caregivers are more vulnerable to stress and physical strain from the caring task because of their own frailty and advanced age.^52^

The findings of the studies reviewed in this article showed that loneliness in older adults when not properly managed can cause serious psychological issues, physical health problems, or even death in extreme cases.^53^ Research evidence found that risk of loneliness increases with age especially for those over 65 years old.^54^ Loneliness can induce psychological stress-related responses in older people such as depression, anxiety, and low self-esteem^55^ or other health-related illnesses, such as cardiovascular diseases.^53^ Reviewed studies indicated that the main determinants of loneliness are low SES and poor health,^54,56^ and not either having or not having a partner.^53^ Physical health problems were associated with psychological problems and limited financial resources.^57^ Loneliness and depressive symptoms act in synergetic ways, diminishing the health and well-being for older adults.^54^ Furthermore, the problem of loneliness in older people can be ameliorated by helping them to establish quality social relationships and networks.^56^

The quality of the social networks plays a crucial role in older people’s care experiences of psychological and well-being issues. Findings from the reviewed studies suggest that limited access to one’s social network is closely related to psychological problems and loneliness.^58^ A longitudinal aging study in the Netherlands surveyed 607 participants, examining the relationship between the types of care networks accessed by older people and their psychological well-being.^59^ It was observed that there was a higher likelihood of depressive problems in older adults receiving a combination of formal and informal care, as this tends to exacerbate feelings of dependence and a loss of control. Having both access to social networks and quality networks matter in ensuring better coping mechanisms with mental health and psychological well-being.^60^ Social networks are particularly important because of the person’s limited capacity to participate in recreational or social activities due to infirmity issues.^61^

#### Theme 4: Cultural issues

Culture plays an important role in the acceptance of informal care and the desire for independent living. The ability of older people to cope with loneliness can be enhanced by maintaining extensive and supportive cultural family networks, as well as having positive marital relations.^53,55,56^ The findings from a quantitative survey of 3742 Asian and African migrants living in Germany indicated that, having a supportive partner, coupled with co-residing with their children, tends to protect older people from the risk of loneliness.^56^ Furthermore, several studies found that migrant families highly value informal support exchange.^17,56^ One study found the opposite, postulating that older people especially those from Caucasian, non-migrant and higher SES background may not be willing to receive care from family members as they are reluctant to place a burden on them or owing to relationship issues.^20^ Barken’s study further revealed that the educated and economically privileged higher SES migrants prefer receiving formal care where funds permit, or struggle on their own to fill in the gaps left by inadequate funding, to avoid burdening their families with care responsibility.^20^

The provision of informal care by family members to their older relative, including ethnic minorities (in developed countries), is underpinned by a sense of honor, virtue, filial piety, and familism.^17,21^ In other words, for Africans, Asians, and other traditional societies, caring is a natural progression of family relationship and members of the family feel duty bound to look after their loved ones regardless of the challenges that they may face. A qualitative study conducted interviews with 12 participants who were caregivers for people with dementia and examined the motivation behind people with a migrant background to providing care.^17^ The study reported that participants undertook the caregiving role with a sense of pride and honor, knowing they were caring for their older parents. Similarly, a qualitative study involving 23 male participants in Tokyo, Japan concluded that adult sons are as equally motivated by filial piety to care for their older parents, as are their adult sisters.^62^ The study warns that adult sons need extra help including support to keep their employment and sustain their well-being.^62^

Adult children’s preference and decision to provide care for their older mothers or fathers can be underpinned by cultural values. A quantitative study in the United States that examined adult siblings’ division of labor toward the care of their older parents found that generally adult children commit more hours to caring for their older mothers than they do for their older fathers.^63^ Furthermore, adult daughters tend to spend more hours caring for their mothers than they do for their fathers. Similarly, adult sons provide more care to their fathers than they do for their mothers. The study concluded that, overall older mothers, in comparison to older fathers, receive more care from adult children.^63^

#### Theme 5: Elder abuse

Elder abuse can be categorized in 5 forms: physical abuse, psychological abuse, sexual assault, financial exploitation, and neglect.^64^ From a global perspective, elder abuse can be quite discreet and hidden, particularly when it is perpetrated by close family members.^65^ For example, an Australian based study posits that at some point in the older person’s life, contentious issues related to wills and family inheritance often arise.^22^ The study noted that these issues may arise particularly between protecting a family’s potential inheritance for future generations and preserving assets to pay for older person’s residential care accommodation and health care. Such decisions are often made within the family context and never raised outside the family.^66^ The outcome of an unresolved conflict in the family could be that the older family member is not cared for adequately or s/he is at risk of abuse.

Traditionally, in the western cultures, wills have been used as vehicles to transfer wealth within the family and across generations. However, such positions are increasingly being challenged and diminished because of the societal shift in cultural, religious norms, and values. In several cases, in the courts of law, family members have challenged the older persons’ decision to donate to charity instead of making a will in favor of the family.^67^ There are also claims that in cases where the older person makes a will to have their wealth being inherited by a charitable organization rather than family, this has impacted on family relationships, with family caregivers being the most affected psychologically as well as economically, before and after their death.^67^ Caregiving literature shows that the family caregiver’s mental health state can be a risk factor to elder abuse.^68^ This suggests that issues of family inheritance need careful planning to avoid possible risk of psychological or economic impact on the family caregiver, which then can lead to heightened risk of elder abuse.

Some studies of elder abuse suggest that poor mental health state, characterized by signs of anxiety, depression, and mood swings of the family caregiver can often place the care recipient at significant risk of physical and verbal abuse.^64,69^ In some cases, issues of elder abuse need to be linked to psychological factors, as discussed earlier. For example, a cross-sectional study conducted in Spain, examined the relationship between the likelihood of elder abuse care victim with high dependency needs and the caregiver’s poor psychological mental health.^68^ The study interviewed 829 participants which included caregivers and care recipients. Findings from the study suggested that caregivers regarding caring to be a burden were highly likely experiencing anxiety and depression, presenting detrimental challenges to their caring roles.^68^

Globally, major cultural and legal differences exist in the disposal of older people’s estate. For example, in Australia, the law of succession is based on the English common law, which upholds the principle of testamentary freedom in disposing the estate.^70^ Some research found that tensions often arise within families regarding the decision of negotiating the older person’s entry into residential care in relation to the disposal of their assets.^22^ In such cases, moral and cultural values often increase complexity of decision making, often leaving families torn between the decision of how much wealth should be left for intergenerational inheritance and that which goes toward the older person’s care.^22^ When this happens, the older person’s autonomy to decision making is eroded owing to reluctance to go against the family’s wishes upon which they rely upon for care.

Furthermore, in diverse and multi-cultural societies such as Australia, New Zealand, and the United States, many overseas born citizens’ cultures may clash with the English common law on succession.^70^ However, such customary cultural beliefs may not always be acceptable with some children raised in these countries but with parents from Asia or Africa, thereby creating tensions within the family. Research suggests that when tensions from family disputes related to inheritance remain unresolved, it can negatively impact the caregiving support exchange and relationship between older children with their older parents.^67^

#### Theme 6: Legal, ethical, and human rights perspectives

In many countries, laws relating to POA play a pivotal role in the management and safeguarding of older people’s property and personal finances.^23^ There are several challenges and limitations in relation to legal matters, respect for older people’s rights and protection from elder abuse. For example, relevant laws often stipulate the POA appointees’ eligibility and exclusion criteria, including specifying undesirable individuals like those previously convicted of criminal offences involving dishonesties, such as fraud, or insolvency and bankruptcy.^71^ Research, however, also warns of the legal deficiencies, pointing out that the legislation alone cannot be enough to eliminate or reduce incidences of elder abuse.

While POA laws are made with good intentions, research suggests that they can often significantly increase vulnerability to exploitation.^71^ For example, POA can be used as a “vehicle to steal” from the older person especially by those entrusted and appointed with the enduring POA. Evidence notes that many older people trust their spouse, partner or family members to act on their behalf and make decisions in their best interest.^72^ Thus, many older people naturally appoint close family members through the enduring POA. This move may appear logical and natural or even a cultural norm. Unfortunately, some family members who put their hands up to help may be driven by sinister motives such as to steal from their older relative.^72^ In this context, an unethical person granted enduring POA can be tempted to exploit and prey on the older person’s vulnerability and dependence for their care and support.

Older people have rights, including to privacy and confidentiality, as well as being able to make their own choices like other citizens.^73^ As such, in situations where law firms and professionals identify elder abuse issues when working with older people, they first need the consent of the victim to report any such cases to the relevant authorities. Research, however, has shown that lawyers face ethical dilemmas when elder abuse is identified but the older client does not consent to a report being made on the matter.^71^ In such situations, legal practitioners are left in a difficult ethical situation regarding how best to handle such a case by balancing between ensuring protection from harm of the older client and respecting the right to make a choice and maintain autonomy.^71^

Everyone has a right to fair treatment and to make decisions as guaranteed in the Universal Declaration of Human Rights, Article 1 which state that, “All human beings are born free and equal in dignity and rights.”^74^ Older persons, therefore, need to be protected from elder abuse, but without infringing on their rights to dignity and to make choices about personal care. That is the challenging balance. This review has identified several potential human rights violations affecting older people, including service delivery gaps resulting in unmet care needs.^50^ To ameliorate the impact of older people’s unmet care needs, there is a need to develop an understanding of the association of care with issues of legal, ethical, and human rights perspectives to better inform home-based care policies and programs.

## Discussion

In this scoping review we have synthesized research evidence about the role of care dimensions: gender issues, socio-economic status, psychological issues, cultural issues, elder abuse, and legal, ethical and human rights care issues, in relation to care for older persons living in the home. As discussed, globally most older people only get informal care, with a small percentage receiving formal care and/or a mix of both informal and formal care.^75,76^ While these 2 forms of care are critical, the review suggests that care needs to be understood, organized, and provided beyond these 2 perspectives. The delivery of care for older people at home has implications on the greatest number of older persons and hence the focus of this scoping review.

A report from the United Nations noted that globally, nations require immediate policy responses to keep up the challenges caused by the rapid population growth of older persons.^2^ In particular, due to advance health care systems in many high-income OECD countries, the aging population is fast growing and raising concerns about the ability of those countries to provide adequately for the required social protection of older people. These findings have significant implications for policy and practice. In addition, there is an increased pressure on public health care delivery systems because of a growing demand for age-appropriate long-term health care services.^77^ Furthermore, out-migration of younger generations from rural to urban areas with the erosion of informal home-based care resources for older people, particularly in rural areas,^2^ poses significant challenges.

This scoping review has demonstrated that older people’s care in the home is a gendered activity.^10-12,31,33,34^ In many jurisdictions across the world, women provide the bulk of informal care for older people living in the home. For example, in Europe, approximately two-thirds of informal caregivers for older people are women.^78^ An explanation to this finding maybe that women more than men are socialized into the care role throughout their life course.^12^ In addition, in many cultures, societal cultural norms put expectations on spousal caregivers to be able to provide the care necessary for their partners.^19,21^ There is, therefore, a need for social policy reforms to ensure that older persons’ care at home does not lead to exploitation or disadvantage of 1 gender.

What appears clearly problematic are the patterns of oppression, disadvantage, or inequalities of women in relation to men in the older persons’ home care settings. For instance, research shows that women negatively suffer discrimination as a result of providing unpaid informal care. For example, women in comparison to men experience unequal career opportunities, unfair treatment in the workplace, gender wage gap, reduced contributory pension, and income security in later years.^14,31^ In addition, the nurturing and caregiving role that women undertake prohibit them from fully participating in the labor market, thereby exposing them to low socio-economic status and ongoing poor physical and emotional health.^43^ The findings from this scoping review suggest that caregiving-related policies can have an impact in gender disparities. There is also a clear intersection of gender, ethnicity, and SES status as the burden of caring for older persons disproportionately falls on ethnic/migrant low SES women.

Existing evidence shows that there is consistent association of high incidence of loneliness for older people from lower SES backgrounds.^53^ An explanation for this could be that a number of older people from low SES lack financial resources and opportunities (eg, excursions, sports, and church events), to stay in touch with social networks either in-person or virtually.^56^ Our review suggest that social workers and other professionals in caring professions should address the issue of loneliness for older persons in low SES and their families and communities.

Owing to decreased fertility in developed countries, out-migration of younger people (especially from the global south and rural areas globally), low fertility, and childlessness, many older people are missing out on children or grandchildren to look after them. As a result, when the government fails to provide financial resources, they are vulnerable to higher rates of loneliness.^15,53^ Moreover, lower SES groups typically find it difficult to access formal care owing to a myriad of challenges, among them lower literacy/educational levels and a lack of understanding of how the care systems work.^45^ Loneliness can cause serious psychological, mental, or other health problems for older people.^79^ In this review, we found that many older people from collectivist or more family-oriented cultures tend to have strong family bonds which can significantly reduce situations of loneliness.^53^ The limited research available indicates that more debate and further research is needed to investigate what role is played by socio-economic disadvantages in relation to the care experiences of older people and their caregivers. The financial strain is particularly important for those living in developing countries and rural and remote areas globally as demographic trends shift owing to social and economic pressures.

Findings that showed informal caregiving is often associated with poor health and psychological outcomes for the caregiver were consistent in international literature.^16,18,49,51^ There is a need for more empirical evidence examining how the needs of informal caregivers can be consistently met. Like caregivers, older care recipients may also experience psychological issues because of unmet home-based care needs.^50,59^A significant association of psychological distress and chronic pain disorders in older adults is well established in research.^80^ For most older people, optimum provision of aging services is a basic need and a human right. Evidence suggests that basic need or human rights for many older people are not met, and this results in many of them manifesting psychological and depressive symptoms.^59^ This situation can be extremely challenging, particularly for older people as well as their caregivers who may have complex care needs, hail from low SES backgrounds, and live in rural or remote areas. Further research exploring home-based older people and their caregivers’ experiences of psychological issues in informal care service provision in rural and remote areas is needed and would contribute to understanding the challenges and needs in aged care.

Furthermore, in this review, we found issues regarding who cares for the women, who outlive their spouses, when their time of aged care need arrives.^37^ Spousal caregiving studies indicate that women generally have a higher life expectancy, but also experience a higher burden of illness in those late years.^37^ Nonetheless, these findings demonstrate the crucial need to consider this disproportionate care imbalance experienced by women in relation to men, for example when developing home-based care policies.

Giving voice to older persons in making decisions on matters important is a key fundamental human rights issue. Our findings of this review indicate that having an enduring POA for the older person is closely aligned with lower incidents of elder abuse, in particular psychological, physical, and financial abuse.^81^ Despite the facts that this is a noteworthy finding, however, unfortunately this is not always the case as many home-based older people, particularly from lower SES groups, find it considerably difficult to put in place enduring POA.^81^

Furthermore, a human rights approach posits that elder abuse is not exclusively situated within the realms of the older person’s personal relationship^65^ but also on the State’s commitment and responsibility to ensure protection and respect of every citizen’s human rights.^81^ However, this poses ethical dilemmas in some situations where some elderly persons prefer to keep abusive aspects of their lives confidential as part of their right to privacy and confidentiality, whereas practitioners working under mandatory reporting requirements, must report and disclose such information to authorities without obtaining consent.^82^ Such dilemmas suggest that there is a remarkable gap of knowledge on elder abuse^83^ implying that further research is needed on this delicate and sensitive topic, to understand older people and their caregivers’ lived experiences of elder abuse.

While it was not feasible to undertake country wise analysis, a broad trend was discernible. Generally, in most Southern countries there was more reliance on informal care practices, though this is changing in highly urbanized areas, whereas most countries in the Global North were moving more towards formal care systems.

### Implications for Policy, Practice, and Research

It is worth noting that if the 6 caring perspectives are not well-managed, homebased care for older people may have devastating health outcomes for both care recipients and caregivers. Targeted and evidence-informed social policy interventions are needed to protect both the caregivers and the older care recipients from future crisis, including possible breakdown of the home-based care arrangement. Future policy considerations should include prioritizing formal workers performing dual roles—caregiving in their own time as well as paid work, by offering flexible work arrangements and/or financial compensation for time taken to provide homebased care for their loved ones. At the macro level, both policy makers and practitioners need to keep up to date with the international scholarly developments on the influence of care perspectives on individual home-based care circumstances for older people. Where necessary, practitioners should incorporate the new knowledges emerging from the literature into their practice. It is imperative to ensure social policy addresses homebased care equity issues for both older care recipients and their caregivers.

Our review suggests 3 policy implications. Firstly, policies incentivizing informal care should be framed with special consideration to care and its association with issues of gender roles, psychological, cultural, social, and economic status for older people as well as their caregivers, and how these dimensions intersect. Secondly, our findings indicate that the expectation that older people can rely on their family, friends, or social networks when formal care is unavailable might be misplaced and at times unrealistic given the changing nature of families. To fill this gap, community-based caring policies, programs, and services need to be designed. Thirdly, any restrictions to affordable formal care implies a barrier for the most vulnerable groups from low SES who will be affected as they lack the resources to purchase care services using own personal funds. Addressing those barriers should be a policy priority. Practitioners in the aged care sector need to be cognizant of and sensitive to the 6 caring perspectives and to facilitate incorporating them in care planning and practicing for elderly persons by committing adequate resources. Finally, more research is needed to explore the influence of the 6 caring dimensions from the perspectives of caregivers and care recipients. Further, it is imperative to look at lived experiences of care givers and receivers to help inform responsive and effective home-based care policies and programs. More broadly, future research needs to look at the influence of certain factors such as the geographical context, health care systems, gross domestic product, cultural practices, and social protection policies and systems on these perspectives individually and together, and how these facilitate or hinder caring for elderly persons.

### Limitations of the Study

This study has several limitations. As it is based on scoping review, this might overlook specific home-based care complexities facing older people in a given geographical location. Care providing and receiving is a complex phenomenon where relationships play an important role, but this review has not explored that perspective. It is also limited by our subjective interpretation. Moreover, research showed that with scoping reviews, it is almost impossible to extract all the required relevant material from the eligible articles as some may have data presented in inaccessible formats.^27^ Another limitation was in its omission to conduct quality evaluation of the selected studies.

## Conclusion

Despite evidence of progress being made in the 6 caring perspectives, knowledge gaps and practice issues remain. In his scoping review, we have shown that there is much to be learned about the influence of care dimensions and or lack of it and the complex issues associated with them. This may mean that researchers and professionals need to consider these influences in an integrated manner in the context of home-based care policy and practice. In addition, none of the studies in this scoping review focused on how to find a good balance or mix between formal and informal or a mix of the 6 caring perspectives for older people living in their own homes. Purposefully and sensitively mobilizing and using these caring perspectives is likely to enhance the quality of life and wellbeing of older persons.

## Supplemental Material

sj-docx-1-jpc-10.1177_21501319241296618 – Supplemental material for Perspectives of Caring for Older Persons: A Scoping Review Supplemental material, sj-docx-1-jpc-10.1177_21501319241296618 for Perspectives of Caring for Older Persons: A Scoping Review by Ignatius Chida, Manohar Pawar and Ndungi Mungai in Journal of Primary Care & Community Health

## Appendix

**Appendix 1.** Details of Identified Articles for Analysis.

Author (year)	Method/instrument	Country	Sample size
Brandt et al (2023)	Secondary data analysis—Survey of Health, Ageing and Retirement in Europe (SHARE) and English Longitudinal Study (ELSA)	European countries—Northern, Eastern, Western and Southern	N/A
Swinkels et al (2019)	Quantitative/survey	Netherlands	1611 caregivers
Brandao et al (2017)	Qualitative/semi-structured interview	Portugal	71 caregivers
Abellan et al (2017)	Quantitative/survey	2 European countries—Spain and Sweden	45 553
Glauber (2017)	Quantitative/survey	USA	19 122
Rodrigues et al (2017)	Quantitative survey	Austria	44 169 (community-dwelling older people)
Band-Winterstein et al (2019)	Qualitative/semi-structured	Israel	122 primary caregivers
Bertogg and Strauss (2020)	Quantitative/survey	11 European countries (Austria, Belgium, Croatia, Czech Republic, Estonia, Denmark, France, Germany, Greece, Italy, Luxembourg, Poland, Portugal, Slovenia, Spain, Sweden, and Switzerland)	68 231 home based 50+ years old
Fokkema and Naderi (2013)	Quantitative/survey	Germany	3742 (aged between 50 and 79 years)
Deloitte Access Economics (2020)	Secondary data analysis	Australia	N/A
Biggs and Haapala (2013)	Qualitative/textual analysis	Australia	N/A
Schuchman et al (2018)	Secondary data analysis/literature Review	USA	N/A
Kaspiew et al (2016)	Literature review/document analysis	Australia	N/A
United Nations (2015)	Secondary analysis/policy report	United Nations	N/A
Jacobs et al (2019).	Quantitative/survey	USA	38 769 (caregivers and non-caregivers aged 18 years and older)
Grigoryeva (2017)	Quantitative/survey	USA	27 669 (frail elder parents; 4497 married sons)
Heap et al (2018)	Quantitative/survey	Sweden	2089 Oldest old
Broese van Groenou and De Boer (2016)	Secondary data analysis	Netherlands	N/A
New South Wales Government (2020)	Secondary data analysis/policy report	Australia	N/A
Skinner and Sogstad (2022)	Mixed methods—quantitative surveys/telephone interviews	Norway	20 000 caregivers
Quashie et al (2022)	Quantitative/survey	Germany	3023 (50+ years old)
Swinkels et al (2016)	Mixed methods—longitudinal study—secondary data/interviews	Netherlands	3574 care recipients
Lowe (2018)	Qualitative/literature review	Australia	N/A
Moussa (2019)	Secondary data analysis/literature Review	Australia	N/A
Broese van Groenou (2020)	Quantitative/survey	Netherlands	607 older care recipients
Oliva-Moreno et al (2019)	Quantitative/multicenter longitudinal study	Spain	604 adult caregivers
Storey (2020)	Qualitative/literature review	UK	N/A
Mair et al (2016)	Quantitative/survey	UK	39 198 older adults
Prince et al (2015)	Secondary data analysis/literature review	UK	N/A
World Health Organization (2017)	Secondary data/report	WHO report	N/A
Ribeiro et al (2015)	Mixed methods—semi-structured interviews		43 caregivers (**adult children**)
Albuquerque (2022)	Secondary data analysis—Survey of Health, Ageing and Retirement in Europe (SHARE)	European countries—Portugal, Spain, Italy, and Greece	N/A
Vicente et al (2022)	Quantitative/cross sectional questionnaire-based survey	Sweden	11 168 informal carers
del-Pino-Casado (2021)	Qualitative/lit review	United Kingdom	N/A
Chen et al (2019)	Mixed methods—longitudinal survey/interviews	China	1646 older people
Clark and English (2022)	Mixed methods—case study vignettes	USA	N/A
Carmel (2019)	Secondary data analysis	Israel	N/A
Domenech-Abella et al (2017)	Quantitative/survey	Spain	1124 (older adults 50+ years old)
Haberken et al (2015)	Quantitative/survey	11 European countries (Austria, Belgium, Denmark, France, Germany, Greece, Italy, the Netherlands, Sweden, Switzerland, and Spain)	19 147 parent-child dyads.
Barken (2017)	Qualitative/semi-structured	Canada	34 care recipients
van Wezel et al (2016)	Qualitative/interviews	Netherlands	28 caregivers
Pillemer et al (2016)	Qualitative/lit review	USA	N/A
Nguyen et al (2016)	Mixed methods/surveys/interviews	USA	837 Older people
Hansen and Slagsvold (2016)	Quantitative/cross sectional study	Norway	33 832 (older people aged 60-80 from 11 European countries)
Orfila et al (2018)	Mixed methods/interview/online survey	Spain	829 family caregivers
Hodgkin (2014)	Qualitative/lit review	Australia	N/A
Visvanathan et al (2019)	Mixed methods—retrospective cohort study	Australia	178 924 datasets for older people
Luppi and Nazio (2019)	Quantitative/survey	Germany	17 011 older people
Ries (2022)	Qualitative/conceptual analysis	Australia	N/A
Vozikaki et al (2018)	Quantitative/survey	11 European countries (Austria, Belgium, Denmark, France, Germany, Greece, Italy, Netherlands, Spain, Sweden, Switzerland)	5074 home based older people—65+ years old
Reinhard (2015)	Quantitative/survey	USA	1677 caregivers
Ahmad et al (2020)	Qualitative/semi-structured interviews	Netherlands	12 caregivers
Tough et al (2020)	Mixed methods/cross sectional surveys/interviews	Switzerland	118 caregivers
Luchetti et al (2021)	Mixed methods—survey/questionnaires	USA	491 caregivers
Umegaki-Costantini (2020)	Qualitative/in-depth interviews	Japan	23 male caregivers
Wilson et al (2016)	Mixed methods—surveys, in-depth interviews, document analysis	Australia	68 qualitative interviews with will-makers
Wilson and Tilse (2015)	Qualitative/editorial	Australia	N/A
Cascella Carbo and Garcia-Orellan	Qualitative/lit review	Spain	N/A
Andersson and Monin (2017)	Quantitative—secondary data/surveys	USA	7026 care recipients
Brandt et al (2021)	Quantitative/survey	17 European countries (Austria, Belgium, Denmark, France, Germany, Greece, Italy, Netherlands, Spain, Sweden, Switzerland, Portugal, Estonia, Czech Republic, Slovenia, Poland, Luxembourg)	32 928 home-based older adults 50+ years
Cash et al (2013)	Qualitative/lit review	Australia	N/A
Tilse et al (2015)	Quantitative/survey	Australia	2400—young people (18- to 24-year-old), and oldest cohort (75+ old).

Source: Author compiled data from the literature review.
